# Low serum vitamin D is associated with axial length and risk of myopia in young children

**DOI:** 10.1007/s10654-016-0128-8

**Published:** 2016-03-08

**Authors:** J. Willem L. Tideman, Jan Roelof Polling, Trudy Voortman, Vincent W. V. Jaddoe, André G. Uitterlinden, Albert Hofman, Johannes R. Vingerling, Oscar H. Franco, Caroline C. W. Klaver

**Affiliations:** Department of Ophthalmology, Erasmus Medical Center, NA2808, PO Box 5201, 3008 AE Rotterdam, The Netherlands; Department of Epidemiology, Erasmus Medical Center, Rotterdam, The Netherlands; Department of Paediatrics, Erasmus Medical Center, Rotterdam, The Netherlands; Department of Internal Medicine, Erasmus Medical Center, Rotterdam, The Netherlands; Department of Orthoptics and Optometry, Faculty of Health, University of Applied Sciences, Utrecht, The Netherlands

**Keywords:** Myopia, Vitamin D, Axial length, Children

## Abstract

The aim of the study was to investigate the relationship between serum 25(OH)D levels and axial length (AL) and myopia in 6-year-old children. A total of 2666 children aged 6 years participating in the birth-cohort study Generation R underwent a stepwise eye examination. First, presenting visual acuity (VA) and AL were performed. Second, automated cycloplegic refraction was measured if LogMAR VA > 0.1. Serum 25-hydroxyvitamin D [25(OH)D] was determined from blood using liquid chromatography/tandem mass spectrometry. Vitamin D related SNPs were determined with a SNP array; outdoor exposure was assessed by questionnaire. The relationships between 25(OH)D and AL or myopia were investigated using linear and logistic regression analysis. Average 25(OH)D concentration was 68.8 nmol/L (SD ± 27.5; range 4–211); average AL 22.35 mm (SD ± 0.7; range 19.2–25.3); and prevalence of myopia 2.3 % (n = 62). After adjustment for covariates, 25(OH)D concentration (per 25 nmol/L) was inversely associated with AL (β −0.043; *P* < 0.01), and after additional adjusting for time spent outdoors (β −0.038; *P* < 0.01). Associations were not different between European and non-European children (β −0.037 and β −0.039 respectively). Risk of myopia (per 25 nmol/L) was OR 0.65 (95 % CI 0.46–0.92). None of the 25(OH)D related SNPs showed an association with AL or myopia. Lower 25(OH)D concentration in serum was associated with longer AL and a higher risk of myopia in these young children. This effect appeared independent of outdoor exposure and may suggest a more direct role for 25(OH)D in myopia pathogenesis.

**Electronic supplementary material:**

The online version of this article (doi:10.1007/s10654-016-0128-8) contains supplementary material, which is available to authorized users.

## Introduction

In the last decades, the prevalence of myopia has increased dramatically in Asia as well as in the Western world [1–3]. Prevalence estimates are now around 2 % in 6-year-old children with European ethnicity, and 12 % in children of Asian descent [4, 5]. These figures rise to 50 % in young European adults [6] and up to 96 % in students from South Korea [7]. Although myopic refractive error can be corrected optically by glasses, contact lenses, or refractive surgery, the longer axial length (>26 mm) increases the life-time risk of severe visual impairment and blindness due to retinal complications [8]. The basis of myopia is a developmental mismatch between the optical components of the eye [9, 10], of which excessive elongation of axial length (AL) in early youth is the most important [11].

The need to reveal the etiology of myopia and develop preventive measures is urgent from a public health perspective. Associations with genetic risk variants [12, 13] and environmental factors such as time spent outdoors [14–16] and education [4, 12] have been well established [17, 18]. Recent studies reported an association with serum 25-hydroxy vitamin D [25(OH)D] concentration and myopia in adolescents [19, 20]. Whether this reflects the association between outdoor exposure and myopia, or whether vitamin D itself plays a role in the pathophysiology is unclear. Studies investigating the potential relation with vitamin D receptor (VDR) polymorphisms found no consistent relationships [21, 22].

Serum 25(OH)D is derived from multiple sources. Cholecalciferol (vitamin D3) is formed in the skin after sunlight exposure, and also absorbed by the gut after dietary intake of e.g., fatty fish. Ergocalciferol (vitamin D2) results from intake of foods containing yeasts and fungi [23, 24] Both precursors are hydroxylated in the liver into 25(OH)D. Its active metabolite 1,25(OH)_2_D is formed after transformation in the kidney [25] and is distributed to other sites of the body thereafter. In non-supplemented individuals, sunlight exposure is thought to be the main determinant of 25(OH)D [24, 26–28]. The main function of 1,25(OH)_2_D is regulation of calcium and phosphate metabolism in bone tissue and plasma, but it also has metabolic functions in insulin metabolism [29, 30]. In neuronal disease such as cognitive decline and Parkinson disease [31, 32], it can be involved in immune responses [33] and in DNA transcription and methylation [34, 35]. Whether 1,25(OH)_2_D has a direct effect on eye growth is currently unclear.

The aim of this study was to investigate the association between 25(OH)D levels, AL, and the risk of myopia in children at age 6 years in a large population-based study. Additionally, influence of time spent outdoors on these relationships, and vitamin D related genotypes was studied.

## Population and methods

### Study population

This study was embedded in the Generation R Study, a population-based prospective cohort study of pregnant women and their children in Rotterdam, The Netherlands. The complete methodology has been described elsewhere [36, 37]. A total of 4154 children underwent an ophthalmologic examination by trained nurses at the research center at age 6 years and underwent blood withdrawal for serum measurements. The study protocol was approved by the Medical Ethical Committee of the Erasmus Medical Center, Rotterdam (MEC 217.595/2002/20), and written informed consent was obtained from all participants. Research was conducted according to the declaration of Helsinki.

### Assessment of AL and myopia

The examination included a stepwise ophthalmological examination. Step 1 consisted of monocular visual acuity with LogMAR based LEA-charts at 3 meter distance by means of the ETDRS method, and ocular biometry including AL (mm) was measured by Zeiss IOL-master 500 (Carl Zeiss MEDITEC IOL-master, Jena, Germany) per eye; five measurements were averaged to a mean AL [38]. Step 2 was carried out in children with a LogMAR visual acuity of >0.1 in at least one eye and in children wearing prescription glasses, and included performance of automated cycloplegic refraction [Topcon auto refractor KR8900 (Topcon, Japan)] and a complete ophthalmologic work up by an ophthalmologist. Two drops (three in case of dark irises) of cyclopentolate (1 %) were administered at least 30 min before refractive error measurement. Pupil diameter was ≥6 mm at time of the measurement. Spherical equivalent (SE) was calculated as the sum of the full spherical value and half of the cylindrical value in accordance with standard practice, and myopia was defined as SE ≤ −0.5D in at least one eye. Children with LogMAR visual acuity ≤0.1, no glasses or ophthalmic history were classified as non-myopic [39, 40].

### Assessment of 25(OH)D

At a median age of 6.0 y (95 % range 5.6–7.9), nonfasting blood samples were drawn by antecubital venipuncture and stored at −80 °C until analysis. Serum samples were collected in all children on the examination day at the research center. The measurements of 25(OH)D (nmol/L) in the samples (110μmL serum per sample) were DEQAS certified and were conducted at the Endocrine Laboratory of the VU University Medical Center, Amsterdam, The Netherlands between July 2013 and January 2014 [41]. Serum 25(OH)D was measured with the use of isotope dilution online solid phase extraction liquid chromatography–tandem mass spectrometry, the ‘gold standard’ (LC–MS/MS) [42] using a deuterated internal standard [IS: 25(OH)D3-d6] (Synthetica AS, Oslo, Norway). This method is highly sensitive and has been widely used in 25(OH)D studies [43, 44]. The limit of quantitation was 4.0 nmol/L; intra-assay CV was <6 %, and interassay CV was <8 % for concentrations between 25 and 180 nmol/L.

### Questionnaire

Each mother completed a questionnaire regarding the daily life activities of their child. Time spent playing outdoors and time spent watching television was obtained using questions such as “how much time does your child spend outdoors/watching television in the morning/afternoon/evening”. Questions were asked for weekdays and weekend days separately, and answers were multiple choice (never, 0–½, ½–1, 1–2, 2–3, 3–4 h). Total time spent in a week was summed and divided by seven to make an average h/day.

### Genotyping of SNPs in vitamin D pathway

Samples were genotyped using Illumina Infinium II HumanHap610 Quad Arrays following standard manufacturer’s protocols. Intensity files were analyzed using the Beadstudio Genotyping Module software v.3.2.32, and genotype calling based on default cluster files. Any sample displaying call rates below 97.5 %, excess of autosomal heterozygosity (F < mean − 4SD) and mismatch between called and phenotypic gender were excluded. Genotypes were imputed for all polymorphic SNPs from phased haplotypes in autosomal chromosomes using the 1000 Genomes GIANTv3 panel. SNPs located in genes involved in the Vitamin D metabolic pathway were studied for association with AL and presence of myopia; i.e., genes determining serum 25(OH)D levels (GC, DHCR7, CYP2R1), a gene involved in activation of serum 25(OH)D (CYP27B1), the vitamin D receptor gene (VDR), and the gene involved in deactivation of 1,25-(OH)_2_D in mitochondria (CYP24A1). A total of 33 SNPs [21, 45, 46] were tested, and analyses were adjusted for multiple testing using Bonferroni adjusted *P* value 0.05/33, *P* = 0.0015.

### Measurement of covariates

Height and weight of children were measured by trained nurses, and BMI (weight/height^2^) was calculated. Age was determined at the time of the visit. Income was obtained using the questionnaire and was clustered in low income (lowest tertile) and higher income. If income at the time of the visit was not available, income at birth was used. Ethnicity was obtained in the questionnaire, according to standardized criteria employed by ‘Statistics Netherlands’, the official national statistics agency [47], concerning the country of birth of parents and child: (1) if both parents were born in the Netherlands, the ethnicity is Dutch; (2) if one of the parents was born in another country than the Netherlands, that country was considered country of birth; (3) if both parents were born in the same country other than the Netherlands, that country was represented; (4) if the parents were born in different countries outside the Netherlands, then the country of the mother was represented; and (5) if that child and both parents were born in different countries outside the Netherlands, the country of birth of the child was represented. Ethnicity was grouped into European and non-European. To adjust for seasonality, four seasons were formed on basis of the month in which the children participated in the study (Winter: December–February, Spring: March–May, Summer: June–August, Autumn: September–November).

### Statistical analysis

Separate analyses were performed for AL and myopia. Differences in covariates between myopia and children without myopia were tested using logistic regression analysis adjusting for potentially confounding effects of age and gender. The relation between 25(OH)D and AL was investigated using multivariable linear regression analysis; the relation with myopia (SE ≤ −0.5D) was analyzed using multivariable logistic regression analysis, Covariates were only added to the model if they were significantly related with the outcome as well as with 25(OH)D. Three models were tested: model 1 only adjusted for age and gender; model 2 for age, gender, BMI, ethnicity, television watching, family income, and season visiting the research center; model 3 additionally adjusted for time spent playing outdoors. Effect estimates were determined per 25 nmol/L 25(OH)D. Beta’s are presented with SE; Odds Ratios (ORs) with 95 % confidence intervals (95 % CI). Statistical analyses were performed using SPSS version 21.0 for Windows software (SPSS Inc).

## Results

### Demographics

A flow diagram presenting the selection of children for the current analysis is shown in Supplement Figure 1. A total of 2666 children were available for analysis of serum Vitamin D and myopia; 2636 children were available for analysis of serum 25(OH)D and AL. Demographic characteristics are presented in Table 1. Children with myopia were on average somewhat older. Adjusted for age and height, girls had smaller AL than boys but not a lower frequency of myopia. Myopic children had a higher BMI, watched more television, and spent less time outdoors. Myopia occurred more frequently in children of non-European ethnicity.

**Table 1:** Demographic characteristics of study participants in Generation R (N = 2666)

	All N = 2666	No myopia N = 2604	Myopia N = 62	*P* value
Characteristics				
Age (years)	6.12 (0.44)	6.12 (0.44)	6.28 (0.65)	0.001
Sex, female (%)	49.1 (1308)	49.1 (1278)	48.4 (30)	0.99
BMI (kg/m^2^)	16.09 (1.71)	16.07 (1.69)	16.86 (2.14)	0.005
Low family income (%)	28.0 (747)	27.5 (715)	51.6 (32)	<0.001
Axial length (mm)	22.35 (0.7)	22.33 (0.7)	23.14 (0.86)	<0.001
Ethnicity (%)				
European	75.5 (2013)	76.3 (1986)	43.5 (27)	<0.001
Non-European	24.5 (653)	23.7 (618)	56.5 (35)	
Activities daily life				
Time spent outdoors (h/day)	1.59 (1.14)	1.60 (1.14)	1.16 (0.96)	0.003
Watching television (h/day)	1.34 (0.99)	1.33 (0.97)	1.83 (1.48)	0.001

Values are means (SD), or percentages (absolute numbers)

*P* values are corrected for age, gender, height in logistic regression

### Serum 25(OH)D

**Fig. 1:**
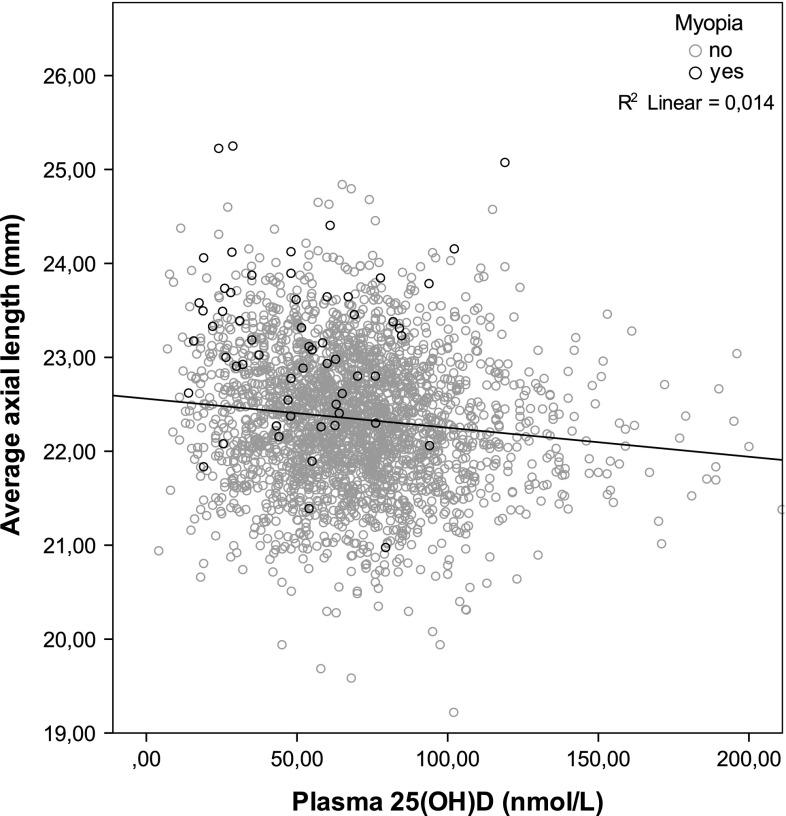
Distribution of axial length as a function of serum level of 25(OH)D in the Generation R cohort

The average serum 25(OH)D in the total study population was lower than the optimal level of 75 nmol/L [23]. Only 37.2 % (1023) of the children reached this optimal level; these were mostly (41.1 %) children who had been examined in summer time (Table 2). Figure 1 shows an inverse relation between serum 25(OH)D and AL for the entire population (*P* < 0.001). Most myopes had high AL and low serum 25(OH)D levels; only 18 % (11/62) of myopic children reached serum levels which corresponded to the optimal level.

**Table 2:** Average serum 25(OH)D (nmol/L) per season in myopic and non-myopic children

Serum 25(OH)D concentration (nmol/L)	N	All	No myopia	Myopia
Child				
All seasons	2666	68.8 (27.5)	**69.2 (27.4)**	**50.2 (24.1)**
Spring	751	60.8 (21.7)	**61.3 (21.6)**	**42.5 (17.5)**
Summer	693	84.2 (28.4)	84.4 (28.4)	69.2(22.6)
Autumn	686	72.9 (26.8)	73.1 (26.8)	63.3 (24.7)
Winter	536	54.7 (23.0)	**55.3 (22.9)**	**36.8 (19.7)**

Values are means (SD)

*P* values are corrected for age, gender, height. *P* values <0.05 are shown in bold

Table 3 shows associations between serum 25(OH)D and AL and myopia. Lower serum levels were associated with higher AL and higher risks of myopia. The estimates remained statistically significant after adjustment for covariates. The effect between serum 25(OH)D and AL remained [beta −0.033 (SE 0.012; *P* 0.02)] after exclusion of myopic children. The association was similar in children of European and non-European descent, but the association with AL in the relatively small non-European group failed to reach statistical significance.

**Table 3:** Multivariate regression analysis of the association between 25(OH)D and axial length and myopia in children at age 6 years

	Model 1: Age and sex adjusted model		Model 2: Multivariate model excluding outdoor exposure		Model 3: Multivariate model including outdoor exposure	
	Association	*P*	Association	*P*	Association	*P*
	N = 2636		N = 2636		N = 2636	
Axial length (mm), beta (SE) of association with 25(OH)D, per 25 nmol/L						
All participants	−0.054 (0.012)	<0.001	−0.043 (0.014)	0.002	−0.038(0.014)	0.007
European ethnicity	−0.051 (0.014)	<0.001	−0.043 (0.016)	0.006	−0.037 (0.016)	0.02
Non-European ethnicity	−0.034 (0.027)	0.20	−0.043 (0.030)	0.16	−0.039 (0.031)	0.20

	Model 1: Age and sex adjusted model		Model 2: Multivariate model excluding outdoor exposure		Model 3: Multivariate model including outdoor exposure	
	Association	*P*	Association	*P*	Association	*P*
	N = 2666		N = 2666		N = 2666	
Myopia, OR (95 % CI) of association with 25(OH)D, per 25 nmol/L						
All participants	0.47 (0.35–0.62)	<0.001	0.63 (0.45–0.89)	0.008	0.65 (0.46–0.92)	0.01
European ethnicity	0.61 (0.39–0.95)	0.02	0.69 (0.42–1.11)	0.13	0.71 (0.44–1.16)	0.17
Non-European ethnicity	0.56 (0.37–0.85)	0.006	0.59 (0.37–0.95)	0.03	0.61 (0.38–0.98)	0.04

The multivariate model for axial length includes adjustment for model 1 and BMI, season of blood withdrawal, ethnicity, television watching, family income. The multivariate model for myopia includes adjustment for model 1 and BMI, ethnicity, television watching, education mother. Outdoor exposure indicates time spent outdoors

### Search for possible explanations

**Fig. 2:**
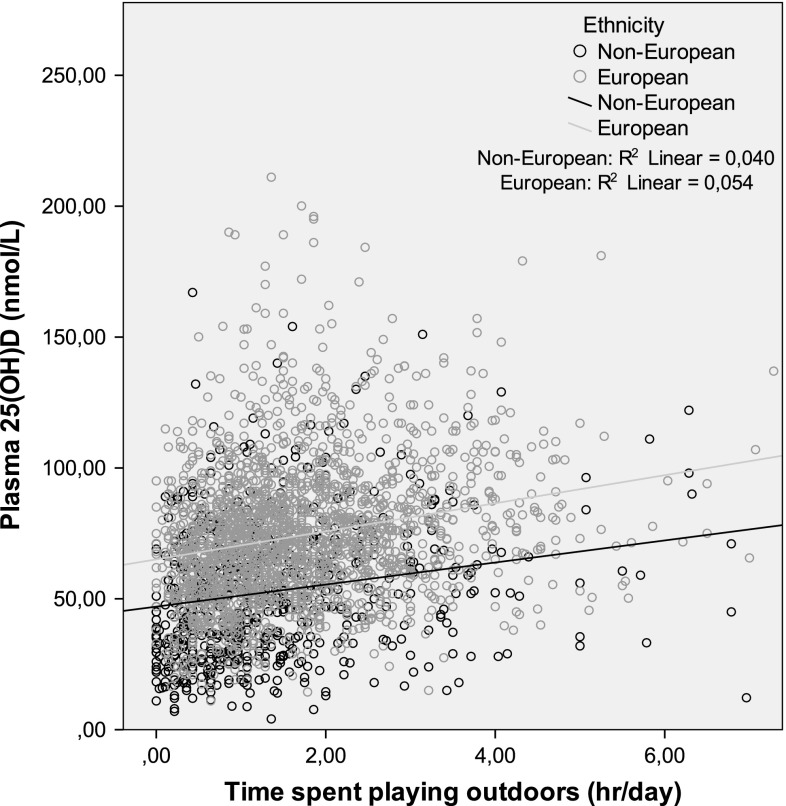
Distribution of serum level of 25(OH)D as a function of time spent outdoors

We hypothesized that our findings could be explained by outdoor exposure. Figure 2 shows the positive relation between time spent outdoors and serum 25(OH)D (Pearson, *P* = < 0.001). Independent of serum 25(OH)D, time spent outdoors (hr/day) was a risk factor for AL [beta −0.034 (SE 0.012; *P* 0.003)]. It was not a significant risk factor for myopia (OR 0.81; 95 % CI 0.61–1.07), possibly due to the small number of myopes. The association between serum 25(OH)D and AL and myopia remained significant after adjustment for time spent outdoors (model 3). We explored possible interactions as well, but there was no significant interaction effect between 25(OH)D, ethnicity or income. Additionally, the association was tested separately in the small subgroup with missing data on time spent outdoors. The effect was similar to the effect in the group with data.

To investigate a possible genetic association between Vitamin D and eye growth, we studied genes incorporated in the Vitamin D pathway. We considered single nucleotide polymorphisms (SNPs) in genes that determine serum 25(OH)D levels, in genes involved in activation of serum 25(OH)D, in the vitamin D receptor gene (VDR), and in the gene involved in deactivation of 1,25-(OH)_2_D_3_ in mitochondria (CYP24A1) (supplemental Table 1). One SNP (rs2245153) in the CYP24A1 gene showed a significant association with AL (beta 0.039; *P* 0.04) and myopia (OR 1.55; 95 % CI 1.04–2.31), 2 SNPs in CYP24A1 (rs4809959 beta 0.032; *P* 0.04 and rs3787557 beta 0.046; *P* 0.04) and one in the VDR (rs11568820 beta −0.042; *P* 0.03) only showed a significant association with axial length. *P* values were all insignificant after adjustment for multiple testing.

## Discussion

In this cohort study of young children, we found a significant association between serum 25(OH)D levels, AL and myopia. In this study children with lower serum levels of 25(OH)D had longer AL, and those with higher 25(OH)D had a lower risk of myopia (OR 0.65; 95 % CI 0.46–0.92 per 25 nmol/L). The association remained significant after adjusting for outdoor exposure, indicating that these two closely related determinants may have some overlapping as well as separate effects on the development of myopia. Genetic variants in the vitamin D pathway genes appeared not to be related: although SNPs in the VDR and CYP24A1 genes showed some association with AL and myopia, this did not remain after adjustment for multiple testing.

Our study had strengths and weaknesses. Assets were the particularly large study sample, the inclusion of the combination of measurements of AL and myopia, and the correction for many potential confounders. The young age of our study population was a benefit as well as a potential drawback. It allowed for measurements of the determinant very close to the onset of myopia, leaving less room for confounding bias. On the other hand, it hampered the study of large effects as most children did not develop excessive eye growth yet. There were other drawbacks. We performed cycloplegia only in children with a diminished visual acuity. Reports show that our cut off value of LogMAR VA of >0.1 had a 97.8 % sensitivity to diagnose myopia [39, 40]. We therefore think that our approach did not substantially affect the number of myopes in our study, nor biased the observed associations. Finally, as the correlation between serum 25(OH)D level and time playing outdoors was relatively low in our study, our questionnaire may not have fully assessed all time spent outdoors. Not all participants filled in the questionnaire completely and data on time spent outdoors was partially missing. However, association in the sample of children without data on time spent outdoors was similar to the association in those with complete data.

A novel finding of our study was that the increase in AL in children with low 25(OH)D was already present in the physiological range of refractive error, before the onset of myopia. This implies that Vitamin D has a continuous effect on AL, and not only determines the development of myopia. We confirmed that the risk of myopia decreased with increasing 25(OH)D levels (OR 0.65) with each 25 nmol/L. The association between 25(OH)D and axial length was also significant in the European children; but failed to reach significance in the Non-European group due to low statistical power. Correction for time spent outdoors demonstrated some attenuation of the association, but did not explain it entirely. Whether this is due to residual confounding of time spent outdoors or whether Vitamin D is truly causally related with AL and myopia remains an open question. The evidence for a role of time spent outdoors in myopia is available from cross sectional studies, intervention and randomized clinical trials as well as from animal studies [15, 16, 48, 51]. Vitamin D production is triggered by UV-exposure, not by light exposure per se. Animal studies have shown that artificial light, free of UV, can inhibit development of myopia development [48]. This may suggests that outdoor exposure and Vitamin D are independent risk factors for axial elongation and myopia. However, true causality cannot be concluded from a cross sectional study; longitudinal and functional studies are needed to provide more profound evidence.

A few previous studies have investigated the role of serum 25(OH)D in myopia. A South-Korean and an Australian study found a positive association in adolescents and young adults [19, 49]. The ALSPAC study found an association with development of refractive error only for 25(OH)D_2_, not for 25(OH)D_3_ in 15 years old children. A potential drawback of this study was the measurement of refraction without any cycloplegia [50]. Mutti et al. [21] found an association between SNPs in the VDR gene and myopia in a smaller study. We could not validate this association, as none of the Vitamin D related SNPs were significant after adjusting for multiple testing.

Various hypotheses underscribe a function of 25(OH)D in eye growth. One theory focusses on Vitamin D in relation to dopamine. The current view is that light exposure initiates the release of dopamine in retinal amacrine cells [51–53]. The released dopamine appears to influence the function of gap junctions and the size of receptive fields [54], an important determinant of eye growth. Vitamin D is known to influence dopamine metabolism in neurological disorders, such as Morbus Parkinson and restless legs syndrome [55]. In particular in Parkinson, Vitamin D protects against cell death in the substantia nigra of the dopamine secreting neuron [32, 56]. Increased dopamine metabolism [57] was found in the rat brain under influence of vitamin D. In the developing rat brain, Vitamin D was found to upregulate glial derived neurotrophic factor (GDNF) which increases dopamine neurons [58]. Taken together, Vitamin D appears to strengthen the function of dopamine or dopamine secreting cells in neuronal tissues. Whether this also accounts for dopamine secreted by amacrine cells in the retina remains an intriguing question.

Another mechanism may be the regulation of DNA transcription in genes containing vitamin D response elements (VDRE, supplemental figure 2). In this case, the active intracellular 1,25(OH)_2_D binds to VDR binding protein, enters the nucleus, and forms a complex with retinoid X receptor in order to bind to VDRE and initiate transcription. VDREs are located in many genes [59]. It has been shown that retinal cells can metabolize 1,25(OH)_2_D; and this active form of vitamin D may interfere with transcription of genes that promote the myopia signaling cascade [60].

In conclusion, we found that serum levels of 25(OH)D were inversely related to AL, and that low levels increased the risk of myopia. Our data suggest that this relationship may be independent from time spent outdoors. The potential role for 25(OH)D in myopia pathogenesis should be further explored by intervention research and functional studies.

## Electronic supplementary material

Below is the link to the electronic supplementary material.

Supplementary material 1 (DOC 133 kb)

## Abbreviations

AL: Axial length
25(OH)D: 25-Hydroxyvitamin D
VA: Visual acuity
VDR: Vitamin D receptor gene
SE: Spherical equivalent
OR: Odds ratio
