# Supplementary material for: Low serum vitamin D is associated with axial length and risk of myopia in young children
Source: Eur J Epidemiol. 2016 Mar 8;31:491–9. doi: 10.1007/s10654-016-0128-8 (PMC4901111; doi:10.1007/s10654-016-0128-8)
Supplement: Supplementary file 1 — Supplementary material 1 (DOC 133 kb) [file 10654_2016_128_MOESM1_ESM.doc]

**Low serum Vitamin D is associated with axial length and risk of myopia in young children**

J. Willem Tideman^1,2^ MD, MSc, Jan Roelof Polling, BoH^1,5^, Trudy Voortman, MSc^2^, Vincent W.V. Jaddoe^2,3^ MD, PhD, André G. Uitterlinden^2,4^, PhD, Albert Hofman^2^ MD, PhD, Johannes R. Vingerling^1^ MD, PhD, Oscar H. Franco^2^ MD, PhD, Caroline C.W. Klaver^1,2^ MD, PhD

^1^Department of Ophthalmology, ^2^Department of Epidemiology, ^3^Department of Paediatrics, ^4^Department of internal medicine, Erasmus Medical Centre, Rotterdam, the Netherlands, ^5^Department of Orthoptics & Optometry, University of Applied Sciences, Faculty of Health, Utrecht, the Netherlands

**Correspondence:**
Prof. Caroline C.W. Klaver, MD, PhD; Erasmus Medical Center NA2808; PO Box 5201, 3008 AE Rotterdam, the Netherlands.
E-mail: [c.c.w.klaver@erasmusmc.nl](mailto:c.c.w.klaver@erasmusmc.nl)

**Supplemental Table 1**: Association between 25(OH)D related SNPs and axial length and myopia in children at age 6 years

|  |  |  |  |  | **Axial length**  **(N=3,938)** | | |  | **Myopia**  **(N=3,928)** | | |
| --- | --- | --- | --- | --- | --- | --- | --- | --- | --- | --- | --- |
| **Gene** | **rs number** | **A1** | **A2** | **Freq A1** | **Beta (SE)** | | ***P*** |  | **OR myopia (95% CI)** | | |
| **Determinants of serum 25(OH)D level** | | | | | |  | |  | |  |  |
| GC | rs2282679 | T | G | 0.76 | 0.018 (0.019) | | 0.35 |  | 1.01 (0.69 – 1.48) | | |
| DHCR7 | rs7944926 | A | G | 0.41 | 0.001 (0.017) | | 0.95 |  | 0.87 (0.62 – 1.21) | | |
| CYP2R1 | rs10741657 | G | A | 0.64 | 0.010 (0.016) | | 0.53 |  | 0.82 (0.60– 1.11) | | |
| **Activation of 25(OH)D** | | | | |  |  | |  | |  |  |
| CYP27B1 | rs8176345 | C | T | 0.97 | -0.020 (0.045) | | 0.65 |  | 0.82 (0.34 – 1.96) | | |
| CYP27B1 | rs4646536 | A | G | 0.69 | 0.008 (0.017) | | 0.65 |  | 1.25 (0.89 – 1.77) | | |
| **Intracellular vitamin D receptor** | | | | |  |  | |  | |  |  |
| VDR | rs7975232 (ApaI) | C | A | 0.45 | -0.012 (0.016) | | 0.47 |  | 1.10 (0.82– 1.49) | | |
| VDR | rs1544410 (BsmI) | C | T | 0.61 | -0.026 (0.016) | | 0.11 |  | 1.07 (0.79 – 1.47) | | |
| VDR | rs731236 (TaqI) | A | G | 0.62 | -0.032 (0.016) | | 0.05 |  | 1.11 (0.80 – 1.54) | | |
| VDR | rs11568820 (CDX2) | C | T | 0.71 | **-0.042 (0.019)** | | **0.03** |  | 0.71 (0.50 – 1.00) | | |
| VDR | rs2228570 (FOK1) | G | A | 0.65 | 0.052 (0.035) | | 0.14 |  | 1.60 (0.78 – 3.28) | | |
| VDR | rs2239182 | T | C | 0.48 | -0.024 (0.016) | | 0.14 |  | 1.19 (0.88 – 1.61) | | |
| VDR | rs3819545 | A | G | 0.62 | 0.027 (0.016) | | 0.10 |  | 0.78 (0.58 – 1.05) | | |
| VDR | rs2853559 | G | A | 0.63 | 0.001 (0.017) | | 0.97 |  | 0.94 (0.68 – 1.31) | | |
| **Mitochondrial inactivation of 1,25-(OH)_2_D_3_** | | | | |  |  | |  | |  |  |
| CYP24A1 | rs2248359 | C | T | 0.56 | 0.018 (0.016) | | 0.25 |  | 1.22 (0.90 – 1.65) | | |
| CYP24A1 | rs6022999 | A | G | 0.70 | 0.008 (0.019) | | 0.65 |  | 1.10 (0.77 – 1.58) | | |
| CYP24A1 | rs2585428 | C | T | 0.54 | 0.020 (0.016) | | 0.19 |  | 1.27 (0.95 – 1.72) | | |
| CYP24A1 | rs2245153 | T | C | 0.79 | **0.039 (0.019)** | | **0.04** |  | **1.55 (1.04 – 2.31)** | | |
| CYP24A1 | rs2296241 | G | A | 0.47 | 0.026 (0.016) | | 0.10 |  | 1.30 (0.97 – 1.75) | | |
| CYP24A1 | rs4809960 | T | C | 0.77 | 0.019 (0.018) | | 0.29 |  | 1.27 (0.87 – 1.87) | | |
| CYP24A1 | rs4809959 | A | G | 0.49 | **0.032 (0.016)** | | **0.04** |  | 1.25 (0.93 – 1.68) | | |
| CYP24A1 | rs2181874 | G | A | 0.72 | -0.030 (0.018) | | 0.10 |  | 0.98 (0.71 – 1.37) | | |
| CYP24A1 | rs3787557 | T | C | 0.87 | **0.046 (0.023)** | | **0.04** |  | 1.12 (0.70 – 1.81) | | |
| CYP24A1 | rs3787555 | C | A | 0.74 | 0.032 (0.018) | | 0.08 |  | 1.27 (0.88 – 1.82) | | |
| CYP24A1 | rs3787554 | G | A | 0.90 | 0.031 (0.024) | | 0.25 |  | 1.35 (0.76 – 2.39) | | |
| CYP24A1 | rs4809958 | T | G | 0.84 | 0.019 (0.021) | | 0.38 |  | 1.07 (0.70 – 1.62) | | |
| CYP24A1 | rs2762939 | G | C | 0.69 | -0.012 (0.019) | | 0.52 |  | 0.97 (0.68 – 1.39) | | |
| CYP24A1 | rs6068816 | C | T | 0.89 | -0.004 (0.026) | | 0.87 |  | 1.00 (0.62 – 1.62) | | |
| CYP24A1 | rs6127118 | G | A | 0.79 | 0.010 (0.022) | | 0.65 |  | 1.08 (0.70 – 1.68) | | |
| CYP24A1 | rs2209314 | T | C | 0.76 | -0.010 (0.021) | | 0.63 |  | 1.07 (0.70 – 1.64) | | |
| CYP24A1 | rs1570669 | A | G | 0.63 | 0.009 (0.017) | | 0.59 |  | 1.35 (0.97 – 1.86) | | |
| CYP24A1 | rs927650 | C | T | 0.65 | 0.004 (0.016) | | 0.79 |  | 0.74 (0.55 – 1.01) | | |
| CYP24A1 | rs2762934 | G | A | 0.80 | -0.018 (0.020) | | 0.36 |  | 1.04 (0.71 – 1.52) | | |
| CYP24A1 | rs6097807 | A | G | 0.72 | 0.013 (0.018) | | 0.49 |  | 1.38 (0.98 – 1.96) | | |
| CYP24A1 | rs6068810 | G | T | 0.95 | 0.025 (0.035) | | 0.47 |  | 0.90 (0.48 – 1.71) | | |

25(OH)D level. Values are in increase in AL (mm) from linear regression models and odds ratios for myopia (95% confidence interval) from logistic regression models. Models are adjusted for age, gender and 10 principal components. A1 is allele 1 and A2 is allele 2. *P* values <0.05 are shown in bold.

**Supplemental figure 1.** Flowchart participants in analysis of 25(OH)D and axial length at age 6 years

**n = 4,154**

Children with 25(OH)D data available

**n = 1,411 with one or more missing covariates**

Missing covariates

playing outdoors n = 1,182

watching television n = 909

Family income = 338

Maternal education = 179

BMI n = 8

Ethnicity n = 2

**n = 2,636**

Children with axial length measurements and covariates

**n = 2,666**

Children with VA >0.1 LogMAR, a ophthalmologic work up and all covariates


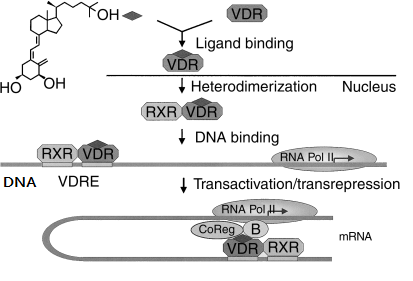


**Supplemental figure 2:** VDR: Vitamin D receptor, RXR: retinoïd X receptor. 1,25(OH)2D signaling is through the VDR in the nucleus. It forms a heterodimer with the RXR receptor. On the DNA strand this complex binds to the VDRE at the promotor of many genes. This results in transactivation of transrepression of genes. (adapted from <http://www.nature.com/ki/journal/v63/n85s/full/4493809a.html>)
